# Adjuvant psychotherapy in early-stage bipolar disorder: study protocol for a randomized controlled trial

**DOI:** 10.1186/s13063-020-04755-8

**Published:** 2020-10-13

**Authors:** Thomas J. Stamm, Julia C. Zwick, Grace O’Malley, Lene-Marie Sondergeld, Martin Hautzinger

**Affiliations:** 1 Department of Clinical Psychiatry and Psychotherapy, Brandenburg Medical School Theodor Fontane, Neuruppin, Germany; 2 Department of Psychiatry and Psychotherapy, Charité University Medicine, Berlin, Germany; 3 Department of Psychology, Clinical Psychology and Psychotherapy, University of Tübingen, Tübingen, Germany

**Keywords:** Bipolar disorder, Group psychotherapy, CBT, RCT, Early intervention

## Abstract

**Background:**

Bipolar disorders are serious illnesses with a chronic course and a high rate of relapse. Typically, bipolar disorders onset during adolescence or early adulthood, with patients experiencing significant personal and social costs as a consequence of their illness. Despite this, to date, there is limited (controlled) evidence regarding the effectiveness of psychotherapy during the critical stages of the disorder (e.g., early onset). Some preliminary studies suggest that targeted, tailored early interventions in particular may improve disease prognosis. The proposed study examines the effectiveness of group psychotherapy on relapse prevention, global adaptive functioning, and neuropsychological functioning in early-stage bipolar disorder.

**Methods:**

In this multicenter randomized controlled trial (RCT), 300 patients with bipolar disorder are randomized to one of two group psychotherapies: Specific Emotional-Cognitive Therapy (SECT; intervention group) or Emotion-Focused Supportive Therapy (EFST; active control group). Each therapy comprises of a total of 48-h sessions (delivered once a month) over a period of 4 months. Assessments take place at baseline (t1); 6 months follow-up, i.e., post-intervention (t2); 12 months follow-up (t3); and 18 months follow-up (t4), whereby 18 months follow-up is the primary time point of interest.

**Discussion:**

The goal of this study is to test the effects of an innovative, specific group therapy relative to an active control condition in terms of rates of relapse, global functioning, and neuropsychological functioning. Pending the outcomes of the trial, it will be possible to establish a firm evidence base for accessible group psychotherapy adjuvant to routine psychiatric care for individuals with bipolar disorder.

**Trial registration:**

USA: ClinicalTrials.gov NCT02506322. Registered on 19 December 2014; Germany: German Clinical Trials Register DRKS00006013. Registered on21 May 2015

## Background

Bipolar disorders are serious illnesses with a chronic course and a high rate of relapse. Compared to unipolar major depressive episodes, patients suffering from bipolar disorder have more than twice as many new episodes (relapses) despite psychiatric care and mood-stabilizing medication. Although the classical description by Kraepelin implies a recurrent disorder with full inter-episodic remission, modern epidemiologic and neuropsychological research has revealed long-term deficits in psychosocial well-being, quality of life, and cognitive functioning [1]. The onset age of bipolar disorders is typically during late adolescence and early adulthood. This is a very sensitive phase for educational, professional, and social development. In addition, this is a critical time in the developmental lifespan characterized by the establishment of one’s personality and often experimentation with oppositional attitudes, chaotic social and sleeping rhythms, and drug use. In bipolar disorder, such activities may challenge compliance and increase the risk of relapse. In particular, young adults (< 30 years) in the early stage of illness experience the highest illness burden with considerable personal costs (e.g., socially, professionally, and personally) and are most vulnerable to death by suicide. Recent meta-analyses of available clinical trials [2, 3] conclude (a) a limited availability of psychotherapy studies in bipolar disorder patients in general and (b) no existing studies focusing on specific subgroups of patients (e.g., early course of disorder and first onset cases). Recently, a staging model implying that bipolar disorder can be classified as a chronic, recurrent disease has been proposed and entails the following stages: asymptomatic (stage 0), prodromal with a low to high-risk profile (stage 1a/1b), onset (stage 2), early stage with occasional relapses and long inter-episode remission periods (stage 3a/b), multiple relapses (3c), and refractory therapeutic course with persistent symptoms and functional deficits (stage 4 [4];).

Applying this model to patients enrolled in psychotherapy studies has shown that the average duration of illness and the number of episodes (on average > 20 episodes) of the examined samples must be assumed to be of stage 3a–c. At the same time, retrospective analyses repeatedly showed that patients of younger age and with few episodes (stages 2–3a) showed significantly better effects of psychotherapeutic interventions [3]. Besides several other RCTs in bipolar depressed patients, we [5] have conducted a RCT with bipolar disorder patients, showing that individual cognitive behavioral therapy (CBT) is superior to individual supportive intervention [6].

Considerations on the stages model of bipolar disorder have therefore also influenced the conceptualization of psychotherapy interventions in recent years: Firstly, two studies examined whether the transition to bipolar disorder could be prevented in the prodromal phase for high-risk individuals [7, 8]. Concerning the late stages of the disease, psychotherapeutic manuals have been developed, specifically targeting functional and cognitive deficits in order to counteract progressive chronicity and functional deficits [9–13]. Within the early stages of the initial disease onset, a number of interventions have been assessed; however, mostly they investigate the influence of psychotherapy on the early course of bipolar disorders with the lack of a control group and with small numbers of cases or without active control conditions (for a review, see [14]. This prompted us to consider the question as to what extent a specific, cognitive behavioral therapy could be superior to a supportive, experience- and emotion-focused intervention in young bipolar patients within the early stage of their disease.

## Methods

### Aims and hypotheses

The general aim of this study is to improve the evidence for psychotherapeutic interventions in relapse prevention for bipolar patients. In order to do so, we wanted to compare two different treatment approaches, to implement new treatment manuals into standard treatment (as there is so far only one available in Germany) and to target patients that are not accessed by the existing practices. Finally, it is our aim to identify clinical and neurobiological predictors for response to psychotherapeutic interventions in bipolar disorder.

To approach these aims, we wanted to test three groups of hypotheses regarding the addition of a specific, innovative psychotherapy (SECT) to psychiatric care in young bipolar patients in the early stage of their disease. Our outcomes are defined as follows: primary—(1) reduced rate of relapse (development of a new affective episode); secondary—(2) reduced missed days at work/school, days spent in hospitals, and health costs; improved medical treatment compliance and social functioning; (3) normalized neurobiological functioning; pre-treatment neural alterations in neurobiological systems will predict post-treatment outcome of SECT, with the greatest improvement in SECT for patients with the most pronounced neural alterations prior to treatment.

### Design and procedure

**Fig. 1:**
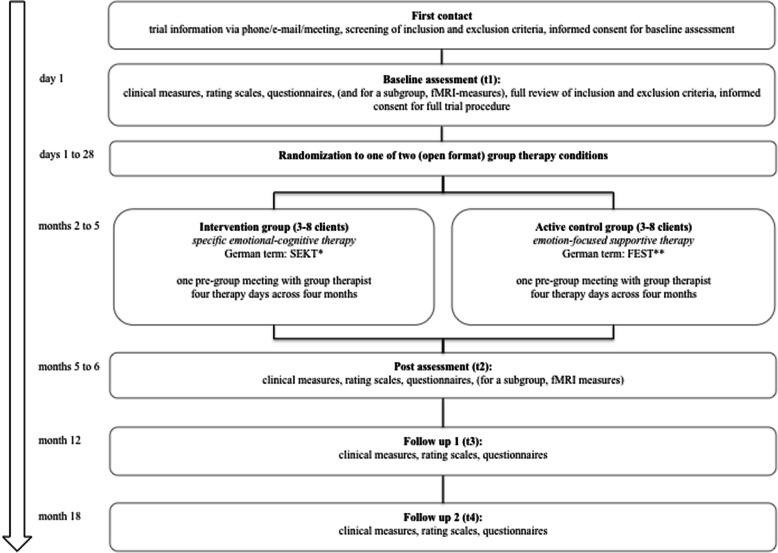
Flowchart summarizing the contact occasions with participants over the course of the study. Notes: *SECT = specific Emotional-Cognitive Therapy for relapse prevention in bipolar disorders (German term: **S**pezifische, **E**motional-**K**ognitive **T**herapie). **EFST = Emotion-Focused Supportive Therapy for relapse prevention in bipolar disorders (German term: **F**ördernde, **E**motionsfokussierte, **S**upportive **T**herapie)

This psychotherapy study is designed as a parallel-group, individually randomized controlled trial (RCT) with blind assessment raters. Patients are screened at baseline (t1) by trained diagnostic clinicians (clinical psychologists or psychiatrists) and enrolled giving their informed consent to the study protocol and meeting full inclusion criteria. Patients are then individually randomized to one of the respective treatment groups. Patients will be immediately informed via phone call about the randomization result by the group therapist they are located to. The group therapist will invite the patient to the next possible group meeting. Groups are ongoing, slow-open groups, admitting new participants when someone is terminated. The intervention takes place over up to 5 months between t1 and t2 and is delivered in four full therapy days of approximately 8 h in small groups up to eight subjects. Patients are then seen at the end of treatment (t2 at month 6) and twice during follow-up (t3 at month 12; t4 at month 18). The flowchart (see Fig. 1) visualizes the study steps.

### Setting and participants

Participants are recruited at 10 university hospital settings providing both in- and outpatient care for individuals with affective disorders within Germany (Eberhard Karls University Tübingen; Charité University Medicine, Berlin; Medical School Brandenburg Theodor Fontane, Neuruppin; University Hospital Carl Gustav Carus Dresden; Ruhr University Hospital Bochum; University Medical Center Hamburg-Eppendorf; University Medical Centre Göttingen; Philipps University Medicine, Marburg; Ludwig Maximilians University Medicine, Munich; Johann-Wolfgang von Goethe University Medicine, Frankfurt). In addition, we will organize local presentations for the public about bipolar disorder, use local media and the clinic homepage to provide information about the study, and use national organization (DGBS) to inform members and their local self-help groups. First, participants are preliminarily telephone screened by a member of the study team to confirm the eligibility criteria. If eligible, the individual will be invited to attend an interview session with a diagnostic clinician to confirm the inclusion criteria.

The inclusion criteria for subjects are as follows:

- 18 to < 35 years of age
- Diagnosis of a bipolar disorder I or II (DSM-V criteria)
- At least one previous episode in the last 2 years
- A minimum of 4 weeks of stable remission since the last affective episode (as measured by the Quick Inventory of Depressive Symptomatology–Clinician Version [QIDS-C] and Young Mania Rating Scale [YMRS], with remission operationalized as QIDS-C < 10 and YMRS < 12)
- In psychiatric care with a personalized medication plan

The exclusion criteria include the following:

- Acute suicidality
- Diagnosis of schizoaffective and/or schizophrenic disorder, substance use disorder (last 6 months), antisocial personality disorder, or a predominant borderline personality disorder
- Currently (or within the last 6 months) undergoing individual psychotherapy.
- Intellectual disability (IQ screening whereby MWT-B < 85)
- A lack of informed consent
- Refusal of psychiatric care including a personalized medication plan (e.g., with mood-stabilizing medication)

All patients continue to receive their routine psychiatric treatment throughout the study, therefore any necessary changes in medication and patient monitoring will occur as appropriate. Patients’ routine psychiatric care continues to be provided within the community, i.e., separate to the study. In some but not all cases, patients also receive care at the host university hospital.

### Randomization

Central randomization either to SECT or to EFST will control for random allocation to both interventions at each research site. For this purpose, an independent statistician developed randomization lists using a centrally computer-generated block-designed randomization procedure stratified by center. Only the principal investigator and his proxy have direct access to the randomization lists. Randomization results are directed at the local study manager.

### Blinding

Throughout the whole study, clinician raters are kept blinded. Results of the individual randomization process will be given only to the local study managers and the group therapists by the principal investigator. Study teams will be trained on how to avoid unblinding during the diagnostic process and team meetings. The statistician who will later analyze the results is as well kept blinded throughout the whole study.

### Intervention

The current study aims to compare two active psychotherapeutic group interventions, which are similar in their format and delivery. Groups include three to eight subjects who meet the outlined inclusion criteria. Each group will meet four times over a 4- to 5-month period for a full day of approximately 8 h on each occasion. All groups are led by purpose-trained, well-experienced psychological psychotherapists or psychotherapists in training on an advanced level (with a minimum of a bachelor’s and master’s degree in clinical psychology and ongoing clinical training). Psychotherapists are trained in both therapeutic interventions in the context of daylong workshops and using videotaped therapy roleplays. All therapists receive extensive feedback from the leading study center in Tübingen prior to delivering a study-relevant treatment group and, as such, are assessed in their competence to administer both interventions. To ensure the quality and coherence of the therapy groups throughout the study, therapists engage in monthly supervision and are required to send video recordings to the lead center for supervision purposes on an ongoing basis (following which written feedback is provided by telephone or email). In the following, the two conditions (i.e., intervention groups) are described in detail.

### Specific Emotional-Cognitive Therapy

The Specific Emotional-Cognitive Therapy (SECT; in German “Spezifische emotional-Kognitive Therapie” [SEKT]) is an innovative, cognitive-behavioral intervention comprising of four modules. Each of the four therapy days focuses on a particular module, which is delivered monthly in the style of a therapy workshop (from 9 am to 5 pm). SECT is a highly structured intervention, and module topics are designed based on their notable clinical relevance (see, for example, [15, 16]) and the empirical evidence base on psychological interventions for bipolar disorder [12, 17–20]. While mindfulness and acceptance (see below) are common interventions across each of the therapy days, the following four distinct modules are delivered across the 4 days, respectively:

1. Psychoeducation, structuring daily life, and life balance
2. Relative normality, detecting early warning signs of an episode, and interpersonal and problem-solving skills
3. Cognitive and meta-cognitive skills
4. Emotion regulation skills

Each treatment day starts with mindfulness meditation, and consolidation of content from the previous therapy day’s content (including review of tasks to be completed at home, e.g., mood and activity diaries and skills development worksheets). The mood and activity diary, for example, is a record kept daily by participants to facilitate observation and learning in relation to mood change, behavior, thoughts, and daily events. Typically, this consistent component of the therapy day is concluded within the first 90 min, and is then followed by proceeding onto the relevant module for the day.

All treatment days are characterized by experiential exercises and the use of vignettes with worksheets and other materials targeting the unique needs of bipolar patients. Although SECT is a structured intervention, participants have an opportunity to work with personal examples relevant to the given topic of the day and are encouraged to exchange challenges and ideas. Therapists follow the common factors in the psychotherapy model proposed by Grawe [21], 2005 [22];. In addition, each patient will be provided with an informational and educational booklet pertaining to bipolar disorder and its treatment (roughly 30 pages in length).

### Emotion-Focused Supportive Therapy

The active control group, Emotion-Focused Supportive Therapy (EFST, in German “Emotionsfokussierte Supportive Therapie” [FEST]), is an interactional, self-help, support group. The concept of EFST was designed for this intervention study. The primary focus of EFST is to provide a platform for group members to exchange personal experiences, engage in self-reflection, express emotions, and provide mutual support. As in SECT, therapists follow the common factors in psychotherapy (Grawe [21], 2005 [22];. EFST therapists are empathetic, help patients to express emotions, give information, and reinforce patients in their self-help strategies and apparent resources. Patients are encouraged to interact with each other and to present opinions and current issues they are facing, as well as to ask one another about shared experiences and coping strategies adopted. In EFST, therapists do not structure meetings or the discussion, but rather facilitate and promote an atmosphere of supportive and friendly group discussion. EFST does not use material, exercises, or give homework such as keeping a mood or activity diary. For psychoeducational reason, each patient is provided with a small booklet about bipolar disorder (e.g., with definitions, statistics, and symptoms) and its pharmacological treatment; however, no specific psychological interventions are provided (e.g., cognitive-behavioral interventions).

### Assessments/measures

Four testing occasions will take place across this longitudinal study, with follow-ups at 5 to 6 months (i.e., immediately post-intervention) and again at 12 and 18 months post-baseline, whereby 18 months follow-up is the primary time point of interest. At baseline, participants’ bipolar disorder is diagnosed by using the Structured Clinical Interview for DSM-IV (structured clinical interview for DSM-IV, SCID 5, German version [23];) with a modified section to assess affective syndromes in line with DSM-V. Current episodes are assessed objectively and subjectively using the Young Mania Rating Scale (YMRS) [24], the Altman Self-Rating Mania Scale (ASRM) [25], and the Quick Inventory of Depressive Symptomatology clinician and self-report forms (QIDS-C, QIDS-SR) [26]. The Global Assessment of Functioning (GAF) [27] and Functional Assessment Short Test (FAST) [28] assess participants’ global social functioning. All measures are administered in the German language and are proven valid and reliable in the German version. The multiple-choice vocabulary intelligence test (“Mehrfachwahl-Wortschatz-Intelligenztest”; MWT) [29] is used as a measure of premorbid IQ. The Longitudinal Interval Follow-up Evaluation (LIFE) (DSM-IV version; developed by Keller et al. [30] and adapted in German for the current study, 2005) is additionally implemented as a key measure in assessing functioning and symptoms in bipolar disorder over time. Following the intervention, therapy satisfaction is assessed using nine items across a 4-point Likert scale using questions such as “The therapy I participated in was of a high quality.” Table 1 gives an overview of the assessment battery as implemented across measurement occasions.

**Table 1:** Schedule of enrollment, interventions, and assessments

	Study period					
	First contact	Baseline/randomization	Intervention	Post-assessment	Follow-up 1	Follow -up 2
**Time point**	***Day 1***	***Days 1 to 28***	***Months 2 to 5***	***Months 5 to 6***	***Month 12***	***Month 18***
**Enrollment**						
**Eligibility screen**	X					
**Informed consent**		X				
**Randomization**		X				
**Interventions**						
***SECT***			X			
***EFST***			X			
**Assessments**						
***SCID***		X				
***LIFE***		X		X	X	X
***YMRS, QIDS-C***		X		X	X	X
***ASRM, QIDS-SR***		X		X	X	X
***GAF, FAST***		X		X	X	X
***MWT-B***		X				
***Therapy satisfaction***				X	X	

This template is in accordance with the Standard Protocol Items: Recommendations for Interventional Trials (SPIRIT) [31]

**Table 2:** Ethical approval bodies and approval reference numbers at participating study sites

Ethics committee	Reference number
Eberhard Karls University Tübingen	235/2014BO1
Charité Berlin	EA1/105/17
Medical School Brandenburg, Neuruppin	Z-01-170614
University Hospital Carl Gustav Carus Dresden	EK 306072015
Ruhr University Hospital Bochum	15-5479
University Medical Center Hamburg-Eppendorf	MC-184/15
University Medical Centre Göttingen	29/2/16
Philipps University Marburg	Studie 30/15
Ludwig Maximilians University Munich	506-15
Goethe University Frankfurt	253/15

### Primary and secondary outcome measures

To examine the effect of the psychotherapy groups, the trial entails one primary outcome measure (relapse) and several secondary outcome measures: time to relapse (LIFE), relapse rate (LIFE), days missed at work/school (record, interview), and social functioning level [32].

Relapse is defined as the development of a new affective (either depression, [hypo] mania, or mixed) episode that meets the diagnostic criteria or results in a hospital admission. Relapse will be assessed every 6 months using the structured LIFE interview (at t2 [end of treatment], t3 [6 months after t2], and t4 [12 months after t2]). The LIFE leads to reliable information regarding the progression and course of illness, time in remission, development of new affective episodes (relapse), and also other mental disorders (co-morbidity). For example, patients are asked at t2, t3, and t4, “The last time we spoke you reported … [description of affective state at the time, e.g., ‘very depressed with problems sleeping’] … How has it been since then? … Since when have you been feeling better/worse?” This is followed with a formal rating based on DSM criteria (“Psychiatric Status Rating”) making note of when the change occurred (“Change Points”) leading to the collection of weekly ratings for the episode. Patients are encouraged to be specific regarding the changes. The interviewer facilitates this by asking questions such as “Was that in November?” and “Did that happen before or after the holiday season?”

Measurements of symptomatology (QIDS-C/SR, YMRS, ASRM), social functioning, and quality of life are assessed to integrate the patient’s everyday perspective (FAST).

Assessment of these outcomes as indicated by changes in these measures over time allows us to address our main hypotheses, i.e., whether SECT versus EFST shows reduced relapse rates, less missed days from work/school, less days spent in the hospital, lower health costs, improved treatment compliance, higher social functioning, and improved neurobiological functioning.

### Sample size calculation

Sample size calculations refer to a log-rank statistic under *α* = 0.05 and *β* = 0.2 (80% power) under the assumptions of (a) an accrual time of 24 months, (b) a total study duration of 48 months with a primary time point of interest at 18 months post-baseline, and (c) a drop-out rate of 12% (the latter based on experiences from previous studies [2, 3, 5]; and relates to the study observation period of 18 months). We define a treatment effect of factor 2 as a relevant effect, i.e., a hazard ratio (HR) of 0.5, for instance, is regarded as a relevant effect. Previous trials have indicated about 4% of the study population under risk (excluding dropouts) will experience a relapse under conventional therapy each month. HR = 0.5 would require 2% per month for the SECT group and a study population of *N* = 360 patients (180 patients per treatment arm) to reach significance under *α* = 0.05 and *β* = 0.2. The study with a proposed *N* = 300 would require HR = 0.46, and the pessimistic scenario of *N* = 200 would have to reach HR = 0.375. Thus, the study with *N* = 300 intended can yield statistical significance if the treatment effect in the SECT group exceeds factor 2. In addition, our sample size calculation is based on the publication by Jahn-Eimermacher et al. [33] which fits—regarding sample sizes for time-to-event data—the requirements of the present study best and results in an identical sample size.

### Statistical analysis

The question as to whether specific psychotherapy can prolong the time until relapse will be addressed by non-parametric survival analyses involving log-rank or Wilcoxon statistics and sensitivity analyses for the ITT (intent-to-treat) sample and the PP (per-protocol, required is participating in at least 75% of treatment sessions) sample. The time-dependent evolution of group-specific hazard rates will be investigated by parametric models addressing the differences in the dynamics of relapse in both intervention arms. Cox regressions will be performed to identify the risk factors for the probability of relapse and time until relapse if the proportional hazard assumption will not be negated. Our planned analyses will allow to identify if the types of participants with missing outcomes look different to those observed outcomes and whether this pattern might be different by arm: treatment arm with no missing primary outcome at primary time point, treatment arm with missing primary outcome at primary time point, control arm with no missing primary outcome at primary time point, and control arm with missing primary outcome at primary time point. Secondary endpoints like symptomatology, days missed at work/school, or social functioning level will be described on a univariate level and enter multivariable group comparisons on the base of an ANCOVA (with the factor kind of intervention (2) and time points (2 to 4) using baseline as a covariate) and involve stepwise variable selection. Based on the results for these covariates, methods such as principal component analysis or latent variable modeling will finally be considered to identify the risk factors for the probability of relapse and time until relapse. We have the intention to use the CONSORT statement for reporting the outcome and final results of this randomized controlled trial.

### Additional analyses

Descriptive statistics showing the measurements over time will be presented whenever appropriate. Serious adverse events and cases of dropouts will be analyzed descriptively.

Interrater reliability will be assessed by independent raters for SCID diagnoses and LIFE ratings as well as for the treatment adherence. As a measure of consistency between measures of the same class, intraclass correlations (ICCs) will be calculated based on independent ratings of three raters each. In order to assess the internal consistency of the treatment adherence scales, Cronbach’s *α* will be measured. Further, treatment adherence will be assessed by comparing the mean scores for each treatment group on the SECT and EFST subscales by calculating *t* tests.

### Quality assurance, blinding, monitoring, and methods against bias

Clinical report forms (CRF) and standard operations procedure [12] have been developed and are available to all sites before starting the research protocol. These forms are updated on a regular basis as per the evolving needs of the study. Education of local study managers and training of diagnostic clinicians (raters) and therapists of both interventions were conducted during the first months at several central workshops. Further central workshops are conducted regularly to train new diagnostic clinicians and therapists and for the purpose of recalibration and monitoring in order to prevent rater and therapist drift. Each rater and therapist first has to successfully complete a certification process before becoming an active study assistant. For the diagnostic clinicians, a certification process is implemented, whereby, firstly, a gold standard is rated; secondly, the diagnostic assessment of a dummy patient is videotaped; and thirdly, one of the first study patients is videotaped. Ratings are approved by two experienced clinicians at the lead study center. Similarly, for therapists, the certification process includes videotaping a dummy group therapy session (30 min) for each condition, which has to be approved by two experienced therapists at the lead study center.

Across the course of the study, raters and therapists are controlled for reliability and adherence by supervising trainers. In regular intervals (once per month), conference calls for raters and therapists each are conducted for supervision to minimize the differences between the centers. For adherence assessment, two time slots (90 min each) of each therapy session will be videotaped. Of each treatment group, three sessions per therapist will be randomly selected and evaluated to measure the adherence to treatment protocol by independent clinicians blind to the diagnosis and treatment condition. To control for reliability, about 30% of SCID and LIFE interviews will also be taped and evaluated by a second clinician.

To ensure the performance according to the study protocol and the quality of the study, monitors visit each site and evaluate using the SOP and CRF at several points (1 during the initiating phase, and at least 1 during accrual time). The monitors check the completeness and plausibility of the data and align the study data to the original data (source data verification). This is accomplished by accessing the original subjects’ charts. The subjects give their permission for this procedure within the informed consent form. The monitor writes a protocol focusing on deviation from the SOP. Each study center is bound to correct any deviation from the SOP as soon as possible.

### Safety aspects

The clinical course of bipolar disorders implies relapses of high risk across the duration of the research. All study contributors assure to observe participant psychopathology carefully at each point of contact. Patients are enrolled in the study during a euthymic phase of the bipolar disorder; however, it is to be expected that depressive and/or manic episodes may occur during the course of the study and the occurrence of the same does not imply study discontinuation. As long as individuals are capable of participating in the group format, they are welcome to participate in the group therapy days. In critical health situations, the group therapist may offer an individual therapy session to provide additional practical and therapeutic support. All additional psychotherapeutic contact with the participants has to be noted down on the study protocol. If required, participants will be supported in liaising with and securing inpatient medical care.

The study follows a strict procedural protocol, and professional observations and communications with patients about their health issues during the study participation are a basis of correct clinical decisions. All contributors are familiar with the protocol and communicate about serious adverse events (SAEs) with the local principal investigator immediately irrespective of whether the event is causally related to the study. SAEs are defined as all fatal or life-threatening events (e.g., suicidal attempt), events that cause hospitalization or longer duration of hospitalization, or events that cause grave disabilities. The local study site is required to document and report SAEs immediately to the central study site. A report is kept in the study file of the participant.

The study has an internal safety board, independent study-related monitors, and an international advisory board (DSMC) as a steering committee. The day-to-day adherence to the study protocol is monitored by the respective principal investigator at each participating institution, who engages in a fortnightly telephone conference where procedural and logistical matters are discussed under the supervision of the head center and principal investigator (PI). In addition, the head center monitors the adherence to the protocol, data assessment, and processing by visiting study centers upon their initiation and yearly throughout the course of the study. At these visits, permission to proceed and requirements to implement necessary changes are discussed both verbally and with written feedback (i.e., via email). The PI then monitors the implementation of necessary changes to meet the protocol requirements. A Data Monitoring Committee (DMC) will be installed consisting of an independent statistician and two supporting clinical psychologists.

### Ethical issues and considerations

In regard to the protection of patients’ data, all patient files are stored in locked cabinets and rooms in line with the university or clinic’s local policies and legal requirements. A code is assigned to each study patient, which is used to file assessment and treatment information in the central server and data management system. Data management and analyses are entirely confidential and under no circumstances includes the name or personally identifying information of an individual patient.

A number of ethical considerations are relevant to the current study. It is notable that the study comprises a group therapy comparison with a targeted intervention (SECT) comparable to an active control group (EFST). Ethically, this is preferable to a classical controlled study, i.e., where patients are assigned to a waiting list, no treatment, or offered treatment as usual. Similar to in SECT, EFST participants receive an information booklet at the start of the intervention and while EFST therapists do not offer the tailored intervention topics such as in SECT, EFST therapists utilize all general therapeutic skills at their disposal and as per their therapy training, e.g., focusing on patients’ emotional experiences, reinforcing positive coping and strengths. Further, EFST therapists do not withhold illness-related information when requested by the patient. Psychoeducation is considered a key component in both therapy conditions.

This study hypothesizes that SECT may be a preferential intervention to EFST. SECT is tailored for bipolar patients based on the existing empirical evidence and clinical factors. However, it should be noted that the intervention in its current form—while considered safe—as of yet lacks empirical support. The monthly workshop format of the intervention is also novel. In other patient groups receiving individual therapy, less frequent sessions appear to impact outcomes negatively (e.g., PTSD and depressed patients, see [34, 35]). However, this may not be the case for patients in remission as has been shown in some studies (e.g., [36]). Indeed, the monthly format is proposed to increase opting-in, particularly for patients living in more rural areas who may have long distances to travel in order to engage in therapy. While therapy is only offered once a month, it should be noted that the “dosage” of therapy is relatively high, with an 8-h day being equivalent to 42-h sessions a week within a month. However, it is ethically notable that participants may not engage in individual psychotherapy external to the study in the 6 months prior to or during the course of the intervention. In instances where patients wish to commence individual psychotherapy during their study participation, patients are free to exercise their right to drop out and are considered to be lost to attrition. Despite this, it cannot be ruled out that patients’ knowledge of these exclusion criteria does not inform their own choice to forgo individual psychotherapy during their participation in the trial.

It is important to acknowledge that as with all RCTs, the current study involved the randomization of patients to either an active or (relatively) passive therapy condition. It is inevitable that clinicians may have an intuition or preference as to which condition may serve the patient’s interests at a given stage in the course of their illness. To eliminate the possibility of such preferential treatment against the interests of the study and research ethics, randomization is blindly completed by the lead study center (who only receives the patient’s age, gender, and number of past episodes to inform the normal distribution of the treatment groups).

## Discussion

To our knowledge, this is the first multicenter RCT that compares two active psychotherapies in the early stage of bipolar disorder. We hypothesize that a specific, educational, cognitive therapy (SECT) will be superior to a supportive (EFST) group therapy regarding the time to relapse into a manic or depressive episode. The second aim of this study is to measure the effect of our interventions on other outcome parameters (social functioning) and further, to identify clinical and neurobiological predictors for successful psychotherapeutic interventions in bipolar disorders.

One limitation of the study might be the relatively new treatment format: Instead of weekly group sessions, we provide participants with four daylong workshops, mostly offered on Saturdays. This facilitates us to access and recruit a broader sample of patients living at a greater distance from the study sites. As the standard care and availability of specialized services for bipolar disorder vary significantly dependent on whether one lives in an urban or rural area, providing a therapeutic service which is accessible to a broader catchment area, is of great importance. Consequently, designing an intervention concept that is accessible to patients living far away from university hospitals and specialized service centers, which tend to be based in urban areas, reflects a unique strength of the study.

Both treatment manuals were finalized shortly before the beginning of the study and hence no data exists on the acceptance of the interventions. A pre-existing CBT manual in our group [15] has been elaborated upon by integrating elements of mindfulness and metacognitive training that had been successfully tested in a pilot study [12] in a cohort of patients with late-stage bipolar disorder. Therefore, it is conceivable that the positive effects in our pilot study cannot be generalized or replicated in a different sample. The EFST treatment manual has been developed closely in line with the one-to-one therapy manual in our previous study [17]. Although the manuals are inspired by those for individual treatment, where they proved to be effective, this does not necessarily mean that we can expect the same results in a group setting. Indeed, we cannot exclude the possibility that the group condition influences the modality of the treatment in a significant way. Furthermore, it is questionable as to whether the recruitment of patients, many of whom are already known to psychiatric services at the various study sites, implies a particularly homogenous sample: Those individuals with bipolar disorder who do not take advantage of professional medical advice or seek out psychotherapeutic support may respond to therapy in a different way.

A further concern is the within-group variability, arising from heterogeneity across the spectrum of bipolar diseases. According to previous research [37], different personality styles within major depressive disorder (MDD) have an influence on how patients respond to therapy and whether they adhere to treatment. By distinguishing two personality types within MDD, the authors have shown that patients with increased self-criticism may be inhibited by structured therapies such as cognitive-behavioral therapy. It is quite conceivable that adherence is similarly influenced by different personality types in bipolar disorder. As we compare two different forms of therapy, one more structured (SECT) than the other (EFST), without assessing the personality traits of patients, we cannot report on to what extent there may be a reciprocal influence. In addition, aspects such as variability of group size and the degree of the structure may play a more significant role and may have a negative impact on the outcome. Another limitation may arise from the uncertainty regarding whether patients, despite awareness of the exclusion criteria regarding concurrent individual psychotherapy parallel to the study, may choose to discretely engage in one-to-one therapy, which could influence the results. Furthermore, we do not assess patients’ preceding therapy experiences, which may result in within-group variability: Someone with an initial maniEFSTation and no therapy experience may respond differently to the intervention from someone with accumulated years of therapeutic care. Finally, although there have been various attempts to create uniform conditions across the various study centers, a multicenter study always bears the risk of a certain variability that cannot be ruled out completely.

One strength of our study lies in the profound experience of the authors in planning and implementing multicenter RCTs in psychotherapy with an emphasis on affective disorders. The second strength is the fact that the study is embedded in the “Network for the Research in Psychiatric Diseases” funded by the Federal Ministry for Education and Research (BMBF). This network merged the most active academic centers for bipolar disorder research in Germany with significant funding to address the research agenda. For instance, a unique electronic study platform has been established for this project. This platform has since enabled the network to improve and simplify data collection and management over the study centers which are distributed nationwide in Germany.

This accumulation of expertise and great logistical support enables us to conduct the biggest RCT in the field of psychotherapy in bipolar disorders to date. On the basis that the study is conducted across multiple sites, we have managed to recruit a large sample size and to create a setting that enables the inclusion of patients from both urban and more difficult-to-reach rural areas. With recruitment of 300 bipolar patients in remission, we are in a position to examine a wide range of scientific questions beyond our primary hypothesis, especially the search for clinical and neurobiological (fMRI) predictors for psychotherapeutic success.

## Trial status

Protocol version number: 2.0 | date: 1 September 2017.

Recruitment started on 01 August 2015; the first group of patients was randomized in November 2015. All study sites are active since October 2017. The approximate date when recruitment will be completed will be 31 December 2019. The follow-ups of study participants will end in January 2020.

## Data Availability

The data is not available until the main results are published.

## Abbreviations

ASRM: Altman Self-Rating Mania Scale
CBT: Cognitive behavioral therapy
DGBS: Deutsche Gesellschaft für Bipolare Störungen
EFST: Emotion-Focused Supportive Therapy (German term: Fördernde Emotionsfokussierte Supportive Therapie)
GAF: Global Assessment of Functioning
HR: Hazard ratio
ITT: Intent-to-treat group
LIFE: Longitudinal Interval Follow-up Evaluation
MWT: Mehrfachwahl-Wortschatz-Intelligenztest
PP: Per-protocol group
PI: Principal investigator
QIDS-C: Quick Inventory of Depressive Symptomatology clinician form
QIDS-SR: Quick Inventory of Depressive Symptomatology self-report form
SAEs: Serious adverse events
SCID: Structured Clinical Interview for DSM
SECT: Specific Emotional-Cognitive Therapy (German term: Spezifische Emotional-Kognitive Therapie)
YMRS: Young Mania Rating Scale
